# A Study of Hydroelectrolytic and Acid–Base Disturbances in MIS-C Patients: A Perspective on Antidiuretic Hormone Secretion

**DOI:** 10.3390/cimb46100681

**Published:** 2024-10-16

**Authors:** Carmen Loredana Petrea (Cliveți), Diana-Andreea Ciortea, Iuliana-Laura Candussi, Gabriela Gurău, Nicoleta Mădălina Matei, Simona-Elena Bergheș, Sergiu Ioachim Chirila, Sorin Ion Berbece

**Affiliations:** 1 Faculty of Medicine and Pharmacy, University “Dunarea de Jos” of Galati, 800008 Galati, Romania; carmen.petrea@ugal.ro (C.L.P.); gabriela.gurau@ugal.ro (G.G.); eo111@student.ugal.ro (S.-E.B.);; 2 Emergency Clinical Hospital for Children “Sf. Ioan”, 800487 Galati, Romania; 3 Emergency Clinical Hospital for Children “Maria Sklodowska Curie”, 041451 Bucharest, Romania; 4 Faculty of Medicine, University Ovidius of Constanta, 900470 Constanta, Romania; sergiu.chirila@univ-ovidius.ro

**Keywords:** MIS-C, pediatric, hydroelectrolytic and acid–base disturbances, ADH secretion, hyponatremia

## Abstract

COVID-19-associated multisystem inflammatory syndrome in children (MIS-C) is a rare autoimmune disorder characterized by a range of polymorphic manifestations, similar to but distinct from other well-known inflammatory syndromes in children. We conducted a retrospective–descriptive study in which we summarized the clinical presentation of, biomarker variations in, and complications occurring in patients diagnosed with MIS-C, admitted to the Emergency Clinical Hospital for Children “Sf. Ioan”, Galati, between July 2020 and June 2024. A total of 36 children met the MIS-C classification criteria according to the WHO-approved case definitions. A total of 41.7% (n = 15) were male and 58.3% (n = 21) were female. The median age of the study group was 4 years (IQR: 1.75–9.25 years). Surgical involvement was suspected in 16.7% (n = 6) of the patients, while 52.8% (n = 19) required intensive care. Clinically, fever was the most common symptom present in 89% (n = 32) of the cases. Gastrointestinal disorders were also common, with 50% (n = 18) presenting with inappetence, 42% (n = 15) with vomiting, and 39% (n = 14) with abdominal pain from admission, which worsened over time. Paraclinically, all patients exhibited signs of inflammation, and 86.1% (n = 31) had hydroelectrolytic and acid–base imbalances. The median hospital stay was 10 days (IQR: 7–12 days), with a stagnant clinical course in most cases. The inflammatory mechanisms in MIS-C, which can affect the secretion of antidiuretic hormone (ADH), were correlated with hydroelectrolytic disturbances and may lead to severe complications. For this reason, it is imperative to evaluate hydroelectrolytic disorders in the context of MIS-C and use diagnostic and prognostic biomarkers to develop effective therapeutic management strategies, ultimately improving the quality of life of affected children.

## 1. Introduction

More than four years after the official declaration of the Coronavirus Disease 2019 (COVID-19) pandemic [1], the impact of Severe Acute Respiratory Syndrome-Related Coronavirus 2 (SARS-CoV-2) on the human body and, in particular, the long-term complications induced by the virus on the immune system are still arousing the interest of the medical world.

The incidence of SARS-CoV-2 infection in the pediatric population was significantly lower than in adults, with frequently mild symptoms and rarely complications or deaths [2]. However, children seemed to be affected to a much greater extent by an immune dysregulation triggered a few weeks after acute COVID-19 acute infection [3].

The first case, which raised the suspicion of immune system damage, was reported by Jones and colleagues [4], on 7 April 2020. Later that month, the Royal College of Pediatrics and Child Health issued an alert about a multisystem inflammatory disorder observed in pediatric patients with current or recent SARS-CoV-2 infection [5]. These children presented with persistent fever, a constellation of symptoms, elevated inflammatory markers, and multiorgan involvement including cardiac, gastrointestinal, renal, hematologic, dermatologic, and neurologic manifestations [6]. Subsequently, the multisystem inflammatory manifestations associated with COVID-19 in children have been defined interchangeably either as pediatric multisystem inflammatory syndrome temporarily associated with SARS-CoV-2 (PIMS-TS) by the European Centre for Disease Prevention and Control (CDC) [7] or as multisystem inflammatory syndrome in children and adolescents temporally related to COVID-19 (MIS-C) by the US Centers for Disease Control and Prevention [8] and the World Health Organization (WHO) [9].

Although many MIS-C patients initially had asymptomatic or mild COVID-19, the syndrome itself was recognized as a dangerous and potentially fatal condition. It was reported that 44.4% of MIS-C patients required admission to pediatric intensive care units (PICUs) due to the rapid onset and the need for special care in critical situations [10,11].

The exact pathophysiology behind the immune dysregulation seen in MIS-C is not fully understood. However, research indicates that a post-infectious, exaggerated immune response occurs following SARS-CoV-2 infection, leading to the release of large quantities of pro-inflammatory cytokines, triggering a “cytokine storm” [12], which can result in severe complications or death. Pro-inflammatory cytokines like interleukin-1β and IL-6 are believed to stimulate the secretion of arginine vasopressin, leading to the syndrome of inappropriate antidiuretic hormone (ADH) secretion (SIADH), which may contribute to hyponatremia in COVID-19 [13]. In critically ill adults during the pre-vaccine COVID-19 era, fluid and electrolyte imbalances—including sodium, potassium, chloride, and calcium disturbances—were frequently observed [14,15]. Among these, hyponatremia emerged as one of the most common electrolyte disturbances, associated with increased morbidity and mortality [16,17]. The severity of COVID-19 in adults has been correlated with serum sodium levels, with hyponatremia serving as a potential surrogate marker for disease severity or the degree of inflammatory response [18].

While the correlation between hyponatremia and COVID-19 is well established in adult populations, studies assessing its prevalence and association with outcomes in pediatric patients with MIS-C are limited [19,20].

## 2. Materials and Methods

A retrospective analysis was conducted on patients admitted to the Emergency Clinical Hospital for Children “Sf. Ioan” in Galati, from 1 July 2020 to 30 June 2024, who were confirmed with MIS-C. The diagnosis of COVID-19-associated multisystem inflammatory syndrome in children (MIS-C) was based on the case definition issued by the Centers for Disease Control and Prevention (CDC) in April 2020 [21] and later updated by the Council of State and Territorial Epidemiologists (CTSE) in October 2022 [22], as approved by the World Health Organization (WHO). The criteria included fever, significant evidence of inflammation (C-reactive protein > 3 mg/dL), dysfunction in at least two organ systems, and either a positive test for current or recent SARS-CoV-2 infection or serologic confirmation of COVID-19 exposure within 4–6 weeks before symptom onset [23].

We collected data that highlighted multiorgan involvement such as clinical symptoms (fever, mucocutaneous, gastrointestinal, cardiac, or hematologic manifestations), and changes in inflammatory markers, and liver/renal parameters. Additionally, we evaluated the patient’s status regarding current or recent SARS-CoV-2 infection. The gathered information was systematically entered into an Excel spreadsheet, where it was analyzed using descriptive statistical methods, and the results were presented both in text form and through graphs.

In pediatric practice, the assessment of biological parameters requires considering different reference ranges based on age. We provided clarifications on these reference ranges, as well as on the equipment and methods used to determine the key biological parameters in our study (Table 1).

To determine the concentration of the electrolytes, such as serum sodium (Na) and serum potassium (K), we employed the “potentiometric method” using Vitros 4600 Chemistry System analyzers (Ortho Clinical Diagnostics, Inc., Rochester, NY, USA).

Serum bicarbonate concentration (ECO_2_) was measured using the “spectrophotometric method” with the ECO slides kit (Ortho Clinical Diagnostics, Johnson & Johnson Company, Wokingham, UK) on the same devices. In our hospital laboratory, serum bicarbonate is also referred to as “alkaline reserve” (AR).

Electrolyte variations, specifically sodium, potassium, and bicarbonate, outside the reference ranges for age and sex were classified as hydroelectrolytic disorders. Hyponatremia and hypernatremia were defined as sodium levels below or above the respective reference ranges for age and sex, while hypokalemia and hyperkalemia were defined similarly for potassium. For ECO_2_ (AR), metabolic acidosis was identified when the value was below the lower limit of the reference range and metabolic alkalosis when the value exceeded the upper limit.

For SARS-CoV-2 RNA detection, the Nimbus Extraction system and CFX 96 amplifier with qRT-PCR were used. The analysis of antibodies (immunoglobulin G and M) against SARS-CoV-2 was conducted using the YHLO-IFLASH 1800 system (YHLO Biotech Co., Ltd., Shenzhen, China) with chemiluminescence.

A total of 36 children and adolescents diagnosed with pediatric multisystem inflammatory syndrome were included in this study. This study received approval from the Medical Council of the Children’s Emergency Clinical Hospital “St. John” under verbal process C399/30.08.2024.

### 2.1. Study Variables

Demographic data, including age, gender, clinical manifestation, and applied therapeutic management, and laboratory findings, such as serum electrolytes, renal and liver function tests, inflammatory markers (D-dimer, serum ferritin, lactate dehydrogenase, and C-reactive protein), were extracted from medical records. This data collection was performed in compliance with the ethical standards of institutional research committees and/or the Declaration of Helsinki (revised in 2013).

For the descriptive analysis, depending on the type of data, we used the median and the interquartile range (for continuous variables), and the number and percentage for qualitative variables were used. The Wilcoxon rank-sum test was used to compare continuous values, while the Chi-square test was used for nominal variables. A *p*-value of less than 0.05 was considered statistically significant.

### 2.2. Inclusion Criteria

The primary inclusion criteria were exclusively children and young people under 18 years of age, confirmed with acute or recent SARS-CoV-2 infection, by specific molecular tests, and diagnosed with MIS-C according to the WHO case definition. This study followed only patients hospitalized in the Emergency Clinical Hospital for Children “Sf. Ioan”, Galati, between 1 July 2020 and 30 June 2024, and for whom parental or legal guardian consent had been obtained. All patients who met the primary criteria, regardless of COVID-19 vaccination status, were included in this study. Recommendations for vaccination against COVID-19 in children over 5 years of age were made by the US Centers for Disease Control and Prevention at the end of 2021 [24]. The cases studied did not specify the vaccination status of the patients against COVID-19.

### 2.3. Exclusion Criteria

Pediatric patients without documented results of interest, with a history of comorbidities such as renal failure/kidney transplantation (estimated glomerular filtration rate < 15 mL/min per 1.73 m^2^ or dialysis-dependent) or heart or other-organ failure were excluded. Children and adolescents undergoing background treatment for chronic conditions or in whom the MIS-C diagnosis was revised during or after hospital admission were also excluded.

## 3. Results

### 3.1. Demographic Data

**Figure 1:**
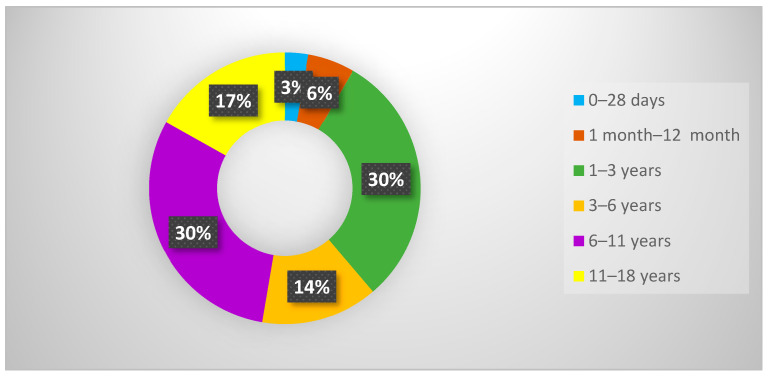
Distribution by age subgroups; patients confirmed with MIS-C.

The retrospective analysis revealed a total of 36 patients diagnosed with multisystem inflammatory disease following SARS-CoV-2 infection who were hospitalized at the Emergency Clinical Hospital for Children “Sf. Ioan”. The patients’ ages ranged from 0 to 15 years, with a median age of 4 years (IQR: 1.75–9.25). The age subcategories showed that the 1–3 year and 6–11 year age groups were the most prevalent, each comprising 30.6% (n = 11) of the total (Figure 1). Among the participants, 58.3% (n = 21) were female, resulting in a female-to-male ratio of 1.4:1. Geographically, 55.5% (n = 20) of the patients were from urban areas.

**Figure 2:**
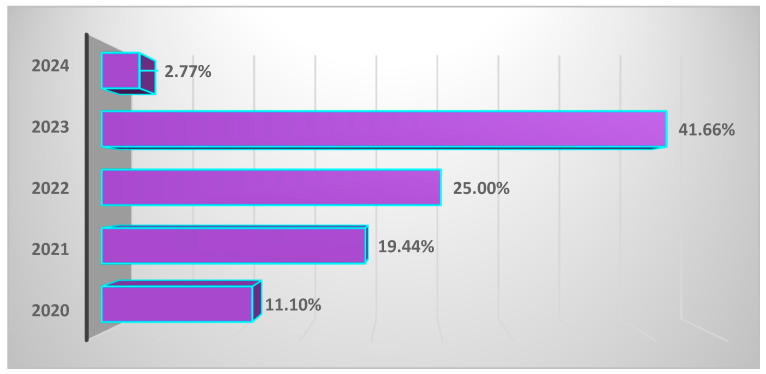
Cases of multisystem inflammatory syndrome in children in the context of COVID-19, reported by year, 2020–2024. Cases were reported as percentages (n = 36, 100%), with each bar indicating the percentage of cases/year.

Relative to the period when this study was conducted, most MIS-C cases were observed in 2023 (41.66%, n = 36) (Figure 2).

Regarding vaccination against COVID-19, the vaccination status was not specified in the cases presented. The first COVID-19 vaccine was administered globally, on 8 December 2020, to a woman in the United Kingdom. Recommendations for administration to children over 5 years of age were made by the US Centers for Disease Control and Prevention (CDC) at the end of 2021 [24]. Considering the data provided, regarding age and period, it can be estimated that at least 30.6% (n = 11) of the pediatric patients were not vaccinated, as MIS-C was described in the period 2020–2021, which is before the recommendation of vaccination against COVID-19. Also, of the cases reported in the period 2022–2024, 41.66% (n = 15) of the children were under 5 years of age.

### 3.2. Constellations of Symptoms and Variations in Biological Parameters in MIS-C

**Figure 3:**
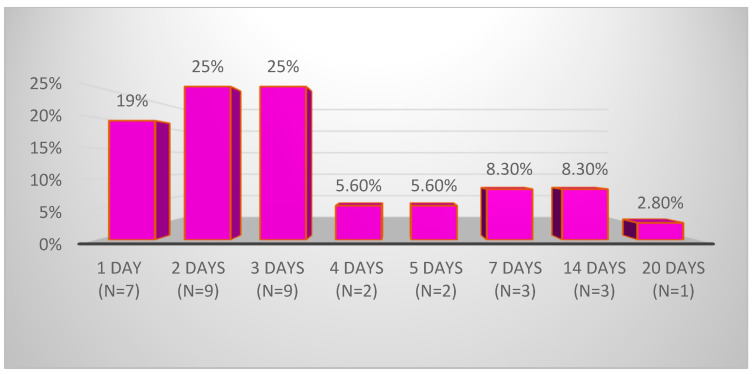
Onset of MIS-C clinical manifestations (y-axis: number of patients in percent [2.8% = 1 patient]; x-axis: number of days from onset of symptoms to hospitalization).

In the studied group, the onset of clinical manifestations, prior to hospitalization, ranged from 1 to 20 days, with a median of 2.5 days (Figure 3). The duration of hospitalization varied from a minimum of 5 days to a maximum of 39 days, with a median of 10.0 days (IQR: 7.0–12.0 days). Among the 36 patients, 55.5% (n = 20) were classified as severe, resulting in a severe-to-moderate ratio of 1.25:1. Additionally, 16.6% (n = 6) were hospitalized or transferred to the Children’s Surgery Department, with clinically and paraclinical supported suspicion of acute appendicitis and/or acute peritonitis. Furthermore, 52.8% (n = 19) of the cases analyzed required intensive care, due to surgical conditions or poor general health.

**Figure 4:**
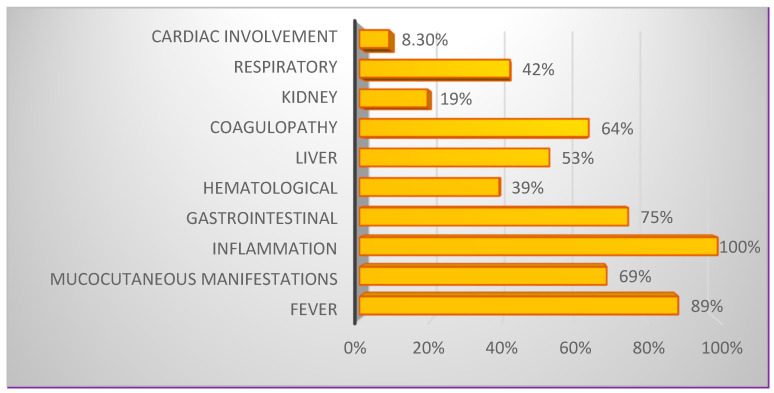
Distribution of the number of patients diagnosed with MIS-C with at least one (or more) symptoms by manifestation categories, in percent (n = 36; 100%).

In our study, MIS-C classification criteria were evaluated according to the case definitions approved by the WHO in December 2022 [23]. These criteria include fever, mucocutaneous manifestations, gastrointestinal symptoms, hematologic changes, marked inflammatory syndrome, and coagulopathy (Figure 4).

Fever was defined as a temperature (T °C) greater than 38.0 °C persisting for at least at least 24 to 48 h. Evaluated mucocutaneous manifestations included petechia, ecchymosis, conjunctival hyperemia, microadenopathies, and localized edema. Gastrointestinal involvement was assessed based on both clinical and paraclinical presentations. The most common gastrointestinal disorders were lack of appetite, vomiting, colicky abdominal pain, and diarrhea, which were present from the onset and exacerbated as the disease progressed. Liver function was evaluated through the measurement of liver enzymes and bilirubin levels. Cardiac involvement was assessed based on rhythm disorders and cardiac contractility (Figure 4). Notably, none of the cases studied presented clinical symptoms of coronary pathologies at admission or during the course of their evolution.

To evaluate the inflammatory syndrome, various paraclinical variables of inflammation were analyzed including C-reactive protein (CRP), erythrocyte sedimentation rate (ESR), ferritin, procalcitonin, and Interleukin 6 (IL-6). C-reactive protein, the most significant marker in MIS-C, showed values ranging from 3.1 to 38.97 mg/dL [Normal Values = 0–0.5 mg/dL], with a median of 15.69 mg/dL (IQR: 4.92–26.72 mg/dL). IL-6, evaluated in 13.9% (n = 5) of the patients, ranged from a minimum of 1.68 ng/dL to 2380 ng/dL [V.N. = 0–0.5 ng/dL]. IL-6 was only assessed in very severe cases and is not considered a routine marker in the evolution of the disease.

The criteria for monitoring hematological status were an absolute lymphocyte count of <10^3^/μL and a platelet count of <150 × 10^3^μL. The lowest recorded value was 0.22 × 10^3^/μL for absolute lymphocyte count and 89 × 10^3^μL for platelet count.

SARS-CoV-2 infection status was assessed by real-time polymerase chain reaction (RT-PCR) from a nasopharyngeal swab for detecting SARS-CoV-2 ribonucleic acid (RNA). At admission, 25% (n = 9) of the patients tested positive, while for the other 75% (n = 27), their immunological status was confirmed through the detection of SARS-CoV-2 immunoglobulin G (Ig G) antibodies.

Two clinical forms of MIS-C were observed in the cohort. Of the 36 cases studied, 55.5% (n = 20) presented severe forms of MIS-C (Table 2). The classification criteria were based on the case definition approved by the WHO. The degree of severity of the disease was determined in terms of the complex of polymorphic manifestations, pathological variations in inflammatory markers, and the number of complications detected.

Table 2 shows categorical variables and symptoms expressed as percentages (%), and numerical variables and biological parameters are expressed as median (IQR). The reference ranges for the biological parameters are presented according to standard clinical guidelines: CRP (0–0.5); ESR (2–12); Procalcitonin (0–0.5); Ferritin (13–68); Leukocytes (4–12); Absolute lymphocytes (3–6.5); Platelet count (150–450); D-dimers (0–0.55); Fibrinogen (150–400); Hemoglobin (11.5–14.5); Erythrocytes (4–5.3); Ionic calcium (3.2–4.2); Creatinine (0.2–0.5); Total protein (6.3–8.2); Aspartate aminotransferase (female—14–36/male—15–59); Alanine aminotransferase (<6 years 15–33: <12 years 18–39; <18 years 17–50); and Urine density (1010–1025). For the biological parameters Sodium, Kalium, and Bicarbonate, the reference ranges are detailed by age and sex in Table 1.

### 3.3. Hydroelectrolytic and Acid–Base Disorders in the Context of MIS-C

Upon admission, 86.1% (n = 31) of the MIS-C patients presented with electrolytic and acid–base (H-E and A-B) imbalances, primarily characterized by altered levels of Sodium (Na), Potassium (K), and Bicarbonate (ECO_2_). The median age of children with electrolyte disturbances was 4 years (IQR: 1.5–9.5 years), and 58% (n = 18) were female (Table 3). The female-to-male ratio in this subgroup was 1.38:1, comparable to the ratio of 1.4:1 observed in the entire MIS-C cohort (n = 36) and 1.5:1 in patients without electrolyte imbalances.

Among the 31 patients with electrolyte disturbances, 58% (n = 18) had severe forms of MIS-C, with a severe-to-moderate ratio of 1.38:1, compared to 1.25:1 in the total group. The highest frequency of electrolyte disturbances in severe cases occurred in the 6–11 years age group (38.8%, n = 7), while in moderate cases, the highest frequency was observed in the 1–3 years age group (38.5%, n = 5) (Table 3).

**Figure 5:**
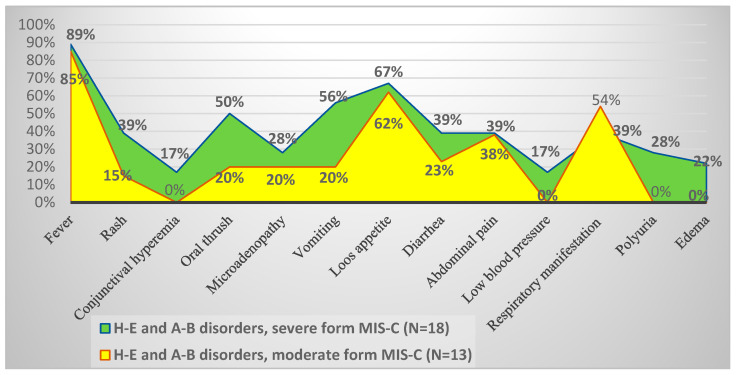
Clinical manifestations, patients with hydroelectrolytic and acid–base disturbances in the context of severe or moderate forms of MIS-C (the x-axis represents the clinical manifestations analyzed; the y-axis indicates the frequency percentages of clinical manifestations in the two groups: severe and moderate forms of MIS-C). The number of patients for each group is shown in the figure.

Several clinical features were observed in patients with electrolyte imbalances in the context of MIS-C (Figure 5). Severe cases were characterized by fever, gastrointestinal symptoms, such as loss of appetite, vomiting, and fewer diarrheal stools. Additionally, patients with severe forms of the disease also presented with urinary flow disturbances and peripheral edema, symptoms that were absent in moderate forms of MIS-C. In contrast, moderate cases had a higher incidence of respiratory symptoms, with loss of appetite being the most frequently observed gastrointestinal issue.

The analysis of biological parameters and demographic characteristics reveal and highlight some differences between the two subgroups of patients with electrolyte disturbances (Table 3).

Table 3 shows categorical variables and symptoms expressed as percentages (%), and numerical variables and biological parameters are expressed as median (IQR). The reference ranges for the biological parameters are presented according to standard clinical guidelines: CRP (0–0.5); ESR (2–12); Procalcitonin (0–0.5); Ferritin (13–68); Leukocytes (4–12); Absolute lymphocytes (3–6.5); Platelet count (150–450); D-dimers (0–0.55); Fibrinogen (150–400); Hemoglobin (11.5–14.5); Erythrocytes (4–5.3); Ionic calcium (3.2–4.2); Creatinine (0.2–0.5); Total protein (6.3–8.2); Aspartate aminotransferase (female—14–36/male—15–59); Alanine aminotransferase (<6 years 15–33: <12 years 18–39; <18 years 17–50); and Urine density (1010–1025). For the biological parameters Sodium, Kalium, and Bicarbonate, the reference ranges are detailed by age and sex in Table 1.

**Figure 6:**
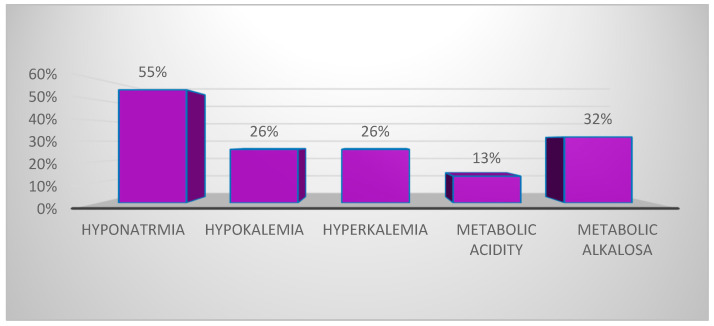
Hydroelectrolytic and acid–base disturbances in MIS-C patients, in percent (the x-axis indicates the different hydroelectrolytic disturbances; the y-axis represents their frequencies expressed as percentages). The percentages are calculated for n = 31, 100%.

Hyponatremia was the most common hydroelectrolytic disorder, observed in 55% (n = 17) of the 31 patients with electrolyte imbalances (Figure 6). Mixed electrolyte abnormalities were observed in 48.3% (n = 15) of the cases, with the most frequent combination being hyponatremia with hypokalemia, hypocalcemia, and metabolic alkalosis, seen in 13% (n = 4) of patients. This was followed by hyponatremia with metabolic acidosis, which was seen in 10% (n = 3) of patients.

#### 3.3.1. Hyponatremia in MIS-C Settings

In patients with hyponatremia, there was no significant gender difference, seeing that 53% of patients were male (Table 3). Sodium levels were less responsive to corrective therapy in severe MIS-C cases compared to moderate forms. A total of 58.8% (n = 10) of all patients with hyponatremia in the context of severe forms of MIS-C required admission to the pediatric intensive care unit (PICU). Hyponatremia in the severe forms of MIS-C had serum sodium values ranging from a minimum of 128 mmol/L to a maximum of 134 mmol/L. In the moderate forms of MIS-C, the lowest value recorded for serum sodium was 130 mmol/L. Of the patients, 76.5% (n = 13) were discharged in improved general condition, 17.6% (n = 3) were discharged in a stationary condition, and one patient was fully cured.

In patients with hyponatremia in the context of severe forms of MIS-C, more than four organ systems were frequently affected. Most often, the affected organ systems were the skin at a frequency of 58.9% (n = 10); the gastrointestinal system, at a frequency of 52.9% (n = 9); and the hematologic and renal systems, each at a frequency of 41.2% (n = 7).

Fever was observed in all hyponatremia cases, with an equal distribution between a fever temperature of >38.5 °C and >39 °C, as well as duration of febrile spikes with 50% lasting > 48 h and 50% lasting > 72 h.

In patients with isolated hyponatremia, gastrointestinal disorders predominated, including loss of appetite, abdominal pain, vomiting, and fever, which were more frequently observed in severe MIS-C cases compared to moderate forms. Although less common, respiratory and mucocutaneous symptoms, such as microadenopathies and edema, and renal manifestations, such as polyuria 11.8% (n = 2), were not excluded.

In patients with hyponatremia, in the context of mixed electrolyte abnormalities, mucocutaneous manifestations, polyuria, and diarrheal stools were more frequently noted in severe MIS-C forms (Table 4).

Table 4 shows categorical variables and symptoms expressed as percentages (%), and numerical variables and biological parameters are expressed as median (IQR). The reference ranges for the biological parameters are presented according to standard clinical guidelines: CRP (0–0.5); ESR (2–12); Procalcitonin (0–0.5); Absolute lymphocytes (3–6.5); and Platelet count (150–450). For the biological parameters Sodium, Kalium, and Bicarbonate, the reference ranges are detailed by age and sex in Table 1.

Paraclinically, in patients with hyponatremia, several aspects were noted. Patients with hyponatremia had a median C-reactive protein (CRP) level of 15.71 mg/dL (7.62–25.67 mg/dL) (Table 4). Hypoproteinemia associated with hyperproteinuria was observed in 41.2% (n = 7) of hyponatremia patients, most commonly in those with mixed electrolyte abnormalities. Moderate thrombocytosis was observed in 17.6% (n = 3), and moderate thrombocytopenia was seen in another 23.5% (n = 4). Pathologic changes in creatinine and urea levels were most commonly associated with metabolic acidosis and hyponatremia, 29.4% (n = 5). Elevated liver enzymes, including aspartate aminotransferase (AST) and alanine aminotransferase (ALT), were liked to mixed hydroelectrolytic and acid–base imbalances. Among those with mixed electrolyte abnormalities, the most frequent syndrome was hyponatremia with metabolic acidosis, 23.5% (n = 4), followed by hyponatremia, hypokalemia, hypocalcemia, and metabolic alkalosis, 17.6% (n = 3), and hyponatremia alone, 17.6% (n = 3).

#### 3.3.2. Potassium Ion Concentration Disorders

In the group of patients with MIS-C and hydroelectrolytic disturbances, hypokalemia was noted in 32.25% (n = 10) cases and hyperkalemia in 25.8% (n = 8) (Table 4). Among patients with hypokalemia, 60% (n = 6) were female. The median age was 7.5 years (IQR: 1.25–9 years), which was lower than the median age for hyponatremia patients. Intensive care was required for seven out of eight patients with hypokalemia, all of whom had severe MIS-C. In three cases, there was suspicion of acute surgical injury. Hypokalemia was more frequently observed in severe MIS-C, with critical values below 2.5 mmol/L being recorded. Fever, lack of appetite, and vomiting were noted only in patients with severe hypokalemia. In 20% (n = 2) of hypokalemia patients, critical potassium values were associated with changes in cardiac activity. For patients with hypokalemia in the context of mixed electrolyte abnormalities, additional mucocutaneous manifestations and disturbances in diuresis were observed (30%, n = 3), particularly in those with combined hypokalemia with hyponatremia (Table 4).

Among inflammatory markers, C-reactive protein had a median value of 24.51 mg/dL (IQR: 9.23–28.71 mg/dL). There was no significant difference between the number of cases with thrombocytopenia and thrombocytosis, the ratio being 1.3:1. The maximum value recorded for platelet count was 645.9 × 10^3^ /µL, while the minimum value recorded was 89 × 10^3^ /µL. In 50% (n = 5) of patients with hypokalemia, critical values of absolute lymphocytes were noted (<0.30 × 10^3^/µL).

Hyperkalemia, which was observed in both clinical forms MIS-C, was present in 75% (n = 6) of the female patients. The median age of patients with hyperkalemia was 1 year (IQR: 0.0–2.5 years), much lower than that of other subcategories. Hyperkalemia was more frequently associated with respiratory symptoms, fever, and gastrointestinal disturbances, the most common being mouth sores and vomiting (Table 4).

In patients with hyperkalemia, a severe inflammatory syndrome was noted, with C-reactive protein reaching a maximum value of 38.97 mg/dL and a median of 22.09 mg/dL (IQR: 4.36–26.71 mg/dL), much higher than the average of the entire group (Table 4).

#### 3.3.3. Bicarbonate Concentration Disorders (ECO_2_)

Among the acid–base imbalances, a decrease in bicarbonate concentration was identified in 13% (n = 4) of patients, while an increased bicarbonate concentration was observed in 32% (n = 10) of the cases.

Metabolic alkalosis was identified in 45% (n = 9) of severe MIS-C cases (n = 20), exclusively in the context of mixed electrolyte and acid–base abnormalities. It was most commonly associated with hyponatremia and hypokalemia in 12.9% (n = 4) of patients and with hyperkalemia in 6.5% (n = 2). The mean age of patients with metabolic alkalosis was 8.5 years (IQR: 4–13 years), with an equal distribution in the 6–11 year and 11–18 year age groups (30%, n = 3 in each group).

Metabolic acidosis was observed only in the context of mixed electrolyte abnormalities: in 12.9% (n = 4) of patients with MIS-C and dyselectrolythemias (n = 31) (Table 4). Male sex dominated with a total of 75% (n = 3) of all patients with metabolic acidosis. It was liked to respiratory symptoms, fever, and gastrointestinal disturbances. Significant variations in inflammatory markers were noted, with C-reactive protein levels showing a median value of 24.74 mg/dL (17.35–31.64 mg/dL) and an ESR of 80 mm/h (57–103 mm/h). Patients with metabolic acidosis required intensive therapy, with 50% (n = 2) responding well to corrective treatment.

## 4. Discussion

This research complements previous studies on MIS-C by evaluating the prevalence and characteristics of hydroelectrolytic and acid–base disturbances reported in severe and moderate forms of COVID-19-associated multisystemic inflammatory syndrome in children.

In our study, dyselectrolytemias were detected upon hospitalization in 86.1% (n = 31) of confirmed MIS-C patients. Regarding the clinical forms, more than half of the patients with electrolyte disturbances presented with severe MIS-C. The clinical context was predominantly characterized by fever and gastrointestinal disturbances, along with mucocutaneous, respiratory, hematologic, cardiac, and renal manifestations (Figure 5). Hyponatremia was the most common electrolyte disturbance, observed in 55% (n = 17) of patients with dyseletrolytemias, followed by alterations in potassium and bicarbonate ion concentrations (Figure 6).

In the case of SARS-CoV-2 infection, studies have shown that the virus can not only cause respiratory illness but also affect the gastrointestinal (GI) tract [25], leading to damage of the normal intestinal mucosa, disruption of normal function, and altered nutrient absorption. Moreover, evidence suggests that SARS-CoV-2 infection is associated with changes in the gut microbiota, contributing to GI disturbances in these patients, and these may lead to dyselectrolythemias. Additionally, mild and self-limited liver damage has been reported in SARS-CoV-2 infection [26].

Similarly, studies have shown that kidney involvement is possible in patients with SARS-CoV-2 infection due to the significant presence of ACE-2 receptors on podocytes and tubular epithelial cells [27]. The high level of these receptors on renal cells, akin to those in the gastrointestinal tract, can result in impaired renal function, leading to fluid and electrolyte disturbances [28].

Based on these studies, the presence of electrolyte disturbances in patients with SARS-CoV-2 infection has been confirmed [29,30], with hyponatremia documented as the most common imbalance and associated with an increased risk of mortality in hospitalized patients [31].

A case–control study also demonstrated that not only hyponatremia, but also electrolyte imbalances, induced by potassium (K) and chloride (Cl) deficiencies were more frequent in COVID-19 patients than in control subjects. Electrolyte disturbances, as a complication of SARS-CoV-2 infection, can have a negative impact by exacerbating acute respiratory distress syndrome (ARDS), increasing the risk of cardiac injury [32], prolonging the length of hospitalization for patients in intensive care, and elevating the risk of death. Thus, electrolyte imbalances due to inadequate sodium and potassium are significant indicators in hospitalized patients with SARS-CoV-2 infection [33].

### 4.1. Hydroelectrolytic Disturbances in Severe Forms of MIS-C

As some hypotheses suggest [34], the median duration of hospitalization for patients with hydroelectrolytic and acid–base disturbances in our study was longer for severe forms than for moderate forms or the overall group: 12 days compared to 7 days and 10 days, respectively. There were no significant differences in sex distribution, indicating that the presence of electrolyte disturbances and the severity of clinical forms were not influenced by this factor. The relatively higher mean age of 5.5 years in the group with severe MIS-C form that presented dyselectrolythemias, compared to 3 years in patients with moderate forms and 4 years in the overall group, was not significant enough to assess it as a factor favoring electrolyte imbalances or MIS-C clinical forms. All three values fall within the same age category (3–6 years), which shares common physiological and pathological features.

We observed associations between dyselectrolythemias and inflammatory markers such as C-reactive protein (CRP), D-dimers, ESR, and ferritin, which were significantly elevated. This may suggest increased inflammation in patients with electrolyte disturbances within the MIS-C context. This aspect was also noted in the few studies that included groups of critically ill children with MIS-C [35,36] in whom significantly elevated values of inflammatory markers such as CRP, ESR, and procalcitonin were found in MIS-C compared to COVID-19 pneumonia [36]. In another study, including pediatric patients with MIS-C, hyponatremia was noted in 80% of cases, with a median value of 130 mmol/L (IQR: 116–135 mmol/L) recorded for serum sodium at admission [35]. Furthermore, the need for intensive therapy in severe cases (94.4%, n = 17) indicates a higher level of inflammation in patients with hydroelectrolytic disorders compared to those without dyselectrolytemias, or even moderate forms, despite being in the same condition. This hypothesis is also confirmed by the *p*-value of 0.01, which indicated a highly statistically significant association between the severity of MIS-C clinical forms and admission to the pediatric intensive care unit (Table 2).

In our studied group, as noted in the literature regarding adults, we identified several aspects of electrolyte imbalances in children with MIS-C.

#### 4.1.1. Hyponatremia: Characteristics and Complications in Patients with MIS-C

In the study cohort, hyponatremia was identified more frequently in patients with severe MIS-C, occurring in 58.8% (n = 10) of cases, compared to those with non-severe MIS-C. The minimum recorded serum level in severe forms was 128 mmol/L, with a median of 134.5 mmol/L (IQR: 133–135 mmol/L) and a median hospitalization duration of 12 days. This suggests that severe hyponatremia may influence the disease course and recovery time. Notably, in severe cases of MIS-C, serum sodium levels were often unresponsive to corrective therapy, contributing to a slower disease progression.

A meta-analysis of MIS-C studies reported hypokalemia in up to 80.8% of patients [37]. This study hypothesized clinical similarities between MIS-C and Kawasaki disease (KD), supported by evidence showing a strong association between hyponatremia and the severity of KD [38].

The incidence of hyponatremia in our cohort, affecting half of the 36 patients, was significantly lower than that reported in the MIS-C meta-analysis but comparable to rates observed in adults with COVID-19. One possible explanation for this discrepancy could be the similarity in the pathophysiology between MIS-C and the inflammatory responses seen in severe COVID-19 cases in adults [19]. In a study involving 9943 adults, Hirsch et al. reported that more than one-third of hospitalized patients had hyponatremia upon admission [19]. Additionally, another European study estimated that the incidence of COVID-19-associated hyponatremia in adults was 16.8% higher compared to a control group of patients without confirmed SARS-CoV-2 infection [17].

Regarding the pathophysiologic mechanisms of hyponatremia in children, in the context of SARS-CoV-2 infection, several causes may contribute to this condition. These include the syndrome of inappropriate antidiuretic hormone secretion (SIADH), decreased effective circulating volume, or extracellular fluid depletion [39]. To diagnose SIADH in these patients, it is essential to measure serum ADH or copeptin levels using hypertonic saline or arginine stimulation. Additionally, urinary and serum osmolarity, along with sodium levels, are critical parameters for determining the mechanism of hyponatremia [40,41].

In general, hyponatremia in children can be categorized into euvolemic, hypovolemic, and hypervolemic hyponatremia [42].

Studies have shown that euvolemic hyponatremia in children with SARS-CoV-2 infection is mainly caused by SIADH, produced by four potential mechanisms: increased cytokine levels [43]; lung tissue and alveolar cell injury; various stimuli such as pain, nausea, and medication; and receiving PPV (positive pressure ventilation) [41].

Increased levels of cytokines, such as IL-6, may directly stimulate non-osmotic release of arginine vasopressin (AVP), the predominant mechanism triggering hyponatremia in pediatric patients with MIS-C [44]. It has also been found that in pediatric patients with COVID-19, pain, nausea, or some drugs stimulate the direct release of AVP [41] and thus trigger hyponatremia. In the case of euvolemic hyponatremia, tertiary adrenocortical insufficiency triggered in the context of severe or stress-induced illness has been considered a rarer but important cause. This results in a biochemical and clinical picture similar to that of SIADH [45].

Another form of hyponatremia in children is characterized by circulating volume depletion that triggers baroreceptor-mediated non-osmotic AVP release [46].

Children with hyponatremia typically experience gastrointestinal issue, with gastrointestinal sodium loss due to vomiting and diarrhea being the most common cause. Studies have also indicated that patients who present with nausea, abdominal pain, or respiratory distress, in the context of SARS-CoV-2 infection, may develop the syndrome of inappropriate antidiuretic hormone secretion (SIADH), due to the non-osmotic release of [47] arginine vasopressin, which is induced by elevated cytokine levels and inflammation. Hyponatremia may also be explained by a significant increase in insensible fluid losses due to pyrexia, tachypnea, or less commonly by renal losses due to renal salt wasting syndrome or diuretic use.

Finally, hypervolemic hyponatremia, observed in the pediatric population with COVID-19, although rare, should not be neglected [42].

Iatrogenic hyponatremia, which is common in hospitalized children and is the consequence of hypotonic intravenous fluid administration, has also been described. The American Academy of Pediatrics has highlighted that children and adolescents with COVID-19 may be at risk of developing excessive secretion of AVP, which impairs free renal excretion of free water and which, combined with the provision of free water in the form of hypotonic fluids, can lead to euvolemic hyponatremia [48].

Similar to the studies published in the scientific literature, our analysis revealed that fever was the predominant manifestation in patients with hyponatremia, accompanied by gastrointestinal disorders, such as vomiting (35.3%, n = 6) and diarrhea (29.4%, n = 5), which were more prevalent in patients with severe forms of MIS-C.

The higher rates of hyponatremia in patients with MIS-C may be attributed to dehydration mechanisms resulting from gastrointestinal sodium loss and inadequate secretion syndrome, both of which occur in the context of significant inflammation due to the pathogenic effect of SARS-CoV-2 in MIS-C. While the association between electrolyte disturbances—particularly hyponatremia—and COVID-19 is well established, in adults, studies examining this relationship in pediatric patients with acute or recent SARS-CoV-2 infection are limited [19,20].

Although the pathophysiology of the occurrence of multisystem inflammatory syndrome is somewhat uncertain, valuable insights have been provided regarding the post-infectious immune dysregulation that lies at the core of the pathogenic mechanisms of SARS-CoV-2 action in the context of MIS-C. The hyperimmune response by T helper cells followed by macrophage activation and the triggering of a potentially life-threatening cytokine storm would be based on the superantigenicity of the virus and its ability to evade phagocytic action [3,49]. At the same time, the overproduction of interleukins (IL-6, IL-1β, and IL-12), LAMP-1, IFNGR2, CD244, immunoglobulins (Ig G, IgA, and IgM), anti-La, and anti-aminoacyl t-RNA synthetase is triggered as a result of the humoral immune response mediated by B1 or B2 cells [49]. All these reactions contribute to the formation and deposition of antigen–antibody complexes in perivascular spaces and a massive increase in vascular permeability.

In another study, a stronger interaction between human T cells and the European strain of SARS-CoV-2 was demonstrated [50], which has implications for the gastrointestinal tract. The higher prevalence of gastrointestinal symptoms, reported to range from 11.4% to 50% [51], has been explained by the significant expression of angiotensin-converting enzyme receptor 2 (ACE-2) in differential erythrocytes. Additionally, gastrointestinal inflammation has been attributed to mucosal chemotaxis, evidenced by increased inflammatory markers [52].

In our study, we could not establish a statistically significant relationship between hyponatremia and markers of inflammation in the clinical forms of MIS-C. However, the median C-reactive-protein (CRP) value was 17.09 mg/dL (IQR: 4.80–27.56 mg/dL) in severe forms, slightly higher than the whole group’s median of 15.71 mg/dL (IQR: 5.04–26.93 mg/dL), with a *p*-value of 0.28. As a sensitive biomarker inflammation and tissue damage, CRP is a nonspecific protein induced by IL-6 in the liver [53]. Therefore, extremely high levels of CRP may be an early predictor of disease severity, potentially preceding the cytokine storm [54].

A statistically significant relationship was found between the duration of hospitalization reported for the clinical forms of MIS-C clinical forms and the variation in serum sodium (*p* < 0.001). With *p*-values < 0.05, we can also estimate that the variation in serum sodium concentration would have a statistically positive correlation with the erythrocyte count and alanine aminotransferase.

A statistically significant relationship between hyponatremia and hypoalbuminemia could not be appreciated, as the study group did not have the required number of patients. However, the presence of hypoalbuminemia associated with hyperalbuminuria in 42.7% (n = 7) of the hyponatremic patients points to a variation in plasma protein concentration in proportion to serum sodium values.

In the context of inflammation, an increase in capillary permeability occurs, resulting in the release of serum albumin into the interstitial space [55]. The decrease in plasma oncotic pressure, induced by hypoalbuminemia, leads to the release of antidiuretic hormone, which may contribute to hyponatremia.

The triggering mechanisms of hyponatremia, coupled with a pronounced inflammatory characterized by very high levels of inflammatory markers, contributed to the impairment of more than three organ systems, particularly noted in cases of mixed electrolyte abnormalities. Moderate thrombocytosis was observed in 17.6% (n = 3) of patients, while moderate thrombocytopenia was noted in another 23.5% (n = 4).

Renal impairment was most frequently noted in cases of mixed electrolyte abnormalities, particularly in patients with hyponatremia accounting for 41.1% (n = 7) of cases. Moderate hepatic complications, indicated by variations in aspartate aminotransferase (AST) and alanine aminotransferase (ALT), were associated with mixed hydroelectrolytic and acid–base imbalances, such as hyponatremia with metabolic acidosis (12.9%, n = 4), hyponatremia combined with hypokalemia, hypocalcemia, and metabolic alkalosis (9.7%, n = 3), and hyponatremia alone (9.7%, n = 3). Major complications included seizures and cerebral edema in 11.7% (n = 2) of the patients with hyponatremia.

#### 4.1.2. Hypokalemia/Hyperkalemia Characteristics and Complications in MIS-C Patients

In patients with hypokalemia, significantly low potassium values were recorded predominantly in severe cases. Serum potassium levels ranged from a minimum of 1.65 mmol/L to a maximum of 3.64 mmol/L. The average duration of hospitalization for patients with hypokalemia was 12.5 days (IQR: 11.5–15 days), which closely mirrored the duration for patients with severe forms of MIS-C and electrolyte disturbances. The mean age for these patients was 7.5 years (IQR: 1.25–9 years), lower than the mean for those with hyponatremia but notably higher than the overall group with other electrolyte disturbances.

The extended length of hospitalization, which exceeded that of the general MIS-C group and the group with severe hydroelectrolytic disturbances, suggests a higher severity of illness in the context of potassium levels that were significantly below the acceptable threshold.

Hypokalemia, likely resulting from disrupted potassium absorption and secretion in the colon, may be due to an aldosterone blockade affecting the mineralocorticoid receptor in the large intestine. This condition has been frequently observed in COVID-19 patients [56], and it exacerbates acute respiratory distress syndrome (ARDS) and increases the risk of cardiac injury [31], especially in individuals with pre-existing lung or cardiac diseases. Additionally, hypokalemia may hint at increased potassium excretion via the kidneys, driven by heightened angiotensin II levels due to the reduced expression of angiotensin-converting enzyme II, following SARS-CoV-2 binding to the ACE 2 receptor [57].

Gastrointestinal disorders in pediatric patients can also lead to potassium ion imbalances. In the context of SARS-CoV-2 infection, disruption of the gut microbiota can impair the normal function of the digestive tract, affecting gut flora and leading to symptoms such as nausea and diarrhea.

Although one of the mechanisms of potassium loss is digestive, in the cases studied, a very low frequency of diarrheal stools, 10% (n = 1) was noted compared to the other symptoms identified in these patients. Symptoms that are not involved in the mechanism of potassium reduction by the gastrointestinal route—such as fever, vomiting, and loss of appetite, which clinically dominated in 100% (n = 10), 70% (n = 7), and 60% (n = 6) of the cases, respectively—were more frequently described.

The presence of polyuria (30%, n = 3), edema (20%, n = 2), and vomiting in patients with hypokalemia, especially in the context of mixed electrolyte abnormalities, such as hyponatremia, suggested the onset of SIADH (syndrome of inappropriate antidiuretic hormone secretion), which likely contributed to complications in these patients.

Furthermore, severe hypokalemia was associated with a median CRP value of 24.51 mg/dL (IQR: 9.23–28.71 mg/dL), significantly higher than the value recorded in the general group or patients with electrolyte disturbances in the context of severe MIS-C. Due to the small sample size, however, no statistical correlation between hypokalemia and inflammatory markers could be established.

In cases with hypokalemia, particularly in severe forms with mixed electrolyte disturbances, more than three organ systems were affected. The most frequent involved systems were the gastrointestinal system (60%, n = 6), followed by the mucocutaneous and renal systems (50% each, n = 5) of all cases with hypokalemia. Cardiac involvement (20%, n = 2) was noted in patients with critically low potassium levels, as well as hematologic (30%, n = 3) and respiratory (20%, n = 2) involvement.

In patients with hyperkalemia, the median age was the lowest at 1 year (IQR: 0–2.5 years), and there was a significant difference between the sexes, showing a female-to-male ratio of 3:1. Elevated serum potassium levels, with values above the critical threshold, were observed in 25% (n = 2) of the patients with hyperkalemia. These elevated levels corresponded with complications in these cases, such as acute renal failure and thrombocytosis.

There were several limitations of this study, primarily due to the small sample size, which limited the ability to perform inferential tests to establish a statistical correlation between hydroelectrolytic disturbances and disease severity or to establish the need for intensive therapy. This also limited the generalizability of the findings to larger populations. Additionally, a further sub-cohort analysis would have allowed us to assess how different SARS-CoV-2 variants (Alpha, Delta, and Omicron) may have influenced the onset of hydroelectrolytic imbalances, potentially by specific subcategories. Another limitation was the retrospective nature of this study, which means that causality cannot be definitively established and some clinical and paraclinical data may be incomplete or inconsistent.

### 4.2. Future Perspectives

The outlook for future research in the field of pediatric and adolescent multisystem inflammatory syndrome in children and adolescents temporally associated with COVID-19 is vast, and expanding the patient cohort is a key direction.

Future studies should include a larger number of cases, thus allowing more advanced inferential tests and statistical analyses to provide a clearer picture of the factors influencing the clinical and paraclinical course of patients.

It would also be of great interest to explore the influence of SARS-CoV-2 variants on the manifestations of MIS-C, given the diversity of viral strains that circulated during the pandemic. Further analyses, including genome sequencing of the virus, could contribute to understanding the relationship between variants and disease severity.

Another important research direction is related to hydroelectrolytic and acid–base disorders. Future investigations could explore in more detail the impact of these disorders on the clinical course of MIS-C patients in order to identify possible correlations with disease severity and long-term prognoses. Longitudinal follow-up of these patients could provide essential data on the risk of late or recurrent complications of the disease.

In addition, further research could investigate how therapeutic interventions applied to patients with hydroelectrolytic disorders influence clinical outcomes, providing clearer guidelines for their optimal management.

All these research directions, which we intend to further investigate through multicenter studies, could contribute to a deeper understanding of MIS-C and optimize therapeutic approaches for improving the long-term outcomes of affected patients.

## 5. Conclusions

This study underscores the critical importance of maintaining electrolyte and acid–base balance to mitigate clinical severity and reduce hospitalization time. Deviation from normal electrolyte levels, as observed in several studies, can lead to complications, especially in patients with SARS-CoV-2 infection.

Our analysis shows that children with MIS-C are particularly prone to hydroelectrolytic and acid–base imbalances, and 86.1% of the group exhibited dyselectrolyte dysfunction including non-severe cases. Hyponatremia was observed in 55% of patients with electrolyte imbalances, consistent with findings from adult COVID-19 studies. Although no significant statistical correlation was found between hyponatremia and inflammatory markers like CRP, ESR, procalcitonin, or IL-6, hyponatremia was more common in severe cases and often approached critical levels.

Inflammatory processes affecting ADH secretion, combined with hydroelectrolytic imbalances, can result in severe renal, neurological, or cardiac complications. Thus, it is crucial to assess these disturbances in MIS-C patients and employ diagnostic biomarkers to accurately evaluate complications and guide effective treatment.

Furthermore, developing a MIS-C severity score that incorporates dyselectrolytemias could enhance disease assessment. A larger, multicenter study would also provide deeper insights into the mechanisms behind hydroelectrolytic disturbances in children with SARS-CoV-2 and offer strategies for more effective management of these imbalances.

## Figures and Tables

**Table 1:** Reference values electrolytes levels by age and gender.

Electrolytes	Age Group	Level Range
Sodium (mmol/L)	<3 years	135–142
	3–<6 years	135–142
	6–<16 years	136–143
	>16 years (Females)	137–142
	>16 years (Males)	137–143
Kalium (mmol/L)	0–<6 years (Females/Males)	3.9–4.6
	>6 years (Females/Males)	3.8–4.9
ECO_2_ (mmol/L)	0–<15 days (Female/Males)	5–20
	15 days–<1 year (Females/Males)	10–24
	1–<5 years (Females/Males)	14–24
	5–<15 years (Females/Males)	17–26
	5–<19 years (Females)	17–26
	5–<19 years (Males)	18–28

**Table 2:** Basic demographic, clinical, and paraclinical characteristics of MIS-C cases.

Variables	Total Batch MIS-C N = 36	Severe Form MIS-C N = 20	Moderate Form MIS-C N = 16	*p*-Value
**Categorial variables N (%)**				
Female sex Male sex	21 (58.3) 15 (41.7)	11 (55) 9 (45)	10 (62.5) 6 (37.5)	0.65
Pediatric surgery	6 (16.7)	4 (20)	2 (12.5)	0.67
Hospitalized in PICU	19 (52.8)	19 (95)	0.0 (0.0)	<0.001
**Numerical variables—Median (IQR)**				
Age (years)	4.0 (1.75–9.25)	5.0 (2.0–9.0)	3.5 (1.0–12.0)	0.85
Initial manifestations (days)	3 (2.0–4.25)	3 (1.25–3.0)	3 (2.0–5.0)	0.40
Hospitalization duration (days)	10.0 (7.0–12.0)	12.0 (10.0–14.5)	7.0 (6.0–9.0)	<0.001
**Symptoms N (%)**				
Fever duration >24/48 h >72 h	27 (75) 5 (13.9)	15 (75) 3 (15)	12 (75) 2 (12.5)	>0.99
Skin rash	7 (19.4)	5 (25)	2 (12.5)	0.43
Conjunctival hyperemia	3 (8.3)	3 (15)	0 (0)	>0.99
Oral thrush	20 (55.6)	11 (55)	9 (56.3)	
Microadenopathy	7 (19.4)	5 (25)	2 (12.5)	>0.99
Vomiting	15 (41.7)	9 (45)	6 (37.5)	0.65
Loss of appetite	18 (50)	8 (40)	10 (62.5)	0.18
Diarrhea	7 (19.4)	5 (25)	2 (12.5)	0.43
Abdominal pain	14 (38.9)	9 (45)	5 (31.5)	0.40
Low blood pressure	3 (8.3)	2 (10)	1 (6.3)	>0.99
Respiratory manifestations	15 (41.7)	7 (35)	8 (50)	0.24
Polyuria	7 (19.4)	6 (30)	1 (6.3)	>0.99
Edema	4 (11.1)	4 (20)	0 (0)	>0.99
**Biological parameters**—**Median (IQR)**				
C-reactive protein [mg/dL]	15.69 (4.92–26.72)	20.91(4.91–27.99)	6.92 (4.84–24.67)	0.28
Erythrocyte sedimentation rate [mm/h]	60.5 (29.5–94)	71 (29.5–94)	50.2 (28.4–95.8)	0.62
Procalcitonine [ng/mL]	1.85 (0.78–6.1)	3.96 (0.81–6.24)	1.76 (0.65–3.87)	0.43
Ferritin [ng/mL]	168 (102–257)	177 (99–257)	135 (103–199)	0.80
Leukocytes [×10^3^**/**µL]	15 (13–22)	16 (12–20)	15 (13–23)	0.75
Absolute lymphocytes [×10^3^**/**µL]	1.65 (1.04–2.55)	1.18 (0.53–2.22)	1.92 (1.58–2.66)	0.034
Platelet count [×10^3^**/**µL]	274 (210–587)	302 (150–611)	265 (235–509)	0.99
D-dimers [mg/L]	2.13 (0.9–8.07)	3 (1–8)	2 (1–3)	0.51
Fibrinogen [mg/mL]	443 (273.75–622)	439 (361–592)	463 (253–652)	0.80
Hemoglobin [mg/dL]	10.67 (10.01–11.57)	10.61 (9.83–11.67)	10.68 (10.3–11.7)	0.53
Erythrocytes [×10⁶/µL]	3.99 (3.79–4.58)	3.90 (3.55–4.22)	4.56 (4.00–5.15)	0.057
Ionic calcium [mg/dL]	4.22 (4–4.36)	4.15 (3.90–4.34)	4.27 (4.10–4.37)	0.19
Creatinine [mg/dL]	0.32 (0.25–0.45)	0.32 (0.21–0.42)	0.32 (0.27–0.50)	0.61
Serum sodium [mmol/L]	135 (133–137)	135 (133–136)	136 (132.5–138)	0.28
Serum potassium [mmol/L]	4.01 (3.66–4.47)	3.93 (3.26–4.32)	4.27 (3.97–4.95)	0.070
Bicarbonate [mmol/L]	22 (18.75–25)	24.5 (18–31)	21.0 (19.5–24)	0.22
Total protein [g/dL]	6.3 (6.1–6.8)	6.30 (5.40–6.80)	6.45 (6.30–7.20)	0.17
Aspartate aminotransferase [U/L]	37 (25.5–52)	38 (30–95.5)	32.1 (23.8–39.7)	0.20
Alanine aminotransferase [U/L]	22 (16–37)	29 (21.7–53.5)	18 (14–21.6)	0.003
Urine density	1013 (1007–1027)	1011.5 (1007–1024.75)	1013 (1008–1020)	>0.99

**Table 3:** Baseline demographic and paraclinical characteristics in patients with severe/moderate forms of MIS-C and hydroelectrolytic and acid–base disturbances.

Variable	MIS-C with H-E and A-B Disorders N = 31	H-E and A-B Disorders, Severe-Form MIS-C N = 18	H-E and A-B Disorders, Moderate-Form MIS-C N = 13
**Categorial variables N (%)**			
Female:male ratio	1.38:1	1.25:1	1.6:1
Pediatric surgery	5 (16.1)	3 (16.7)	2 (15.4)
Hospitalized in PICU	17 (54.8)	17 (94.4)	0 (0.0)
**Numerical variables—Median (IQR)**			
Age (years)	4 (1.5–9.5)	5.5 (2–9)	3 (1–10)
Onset of manifestation (days)	3 (2–4.5)	3 (1.25–3)	3 (2–5)
Hospitalization duration (days)	10 (7–12.5)	12 (11–14.75)	7 (6–8)
**Biological markers**—**Median (IQR)**			
C-reactive protein [0–0.5 mg/dL]	15.71 (5.04–26.93)	17.09 (4.8–27.56)	14.09 (6.54–25.39)
Erythrocyte sedimentation rate [2–12 mm/h]	66 (30–93.4)	71 (30.75–91.25)	55 (30–93)
Procalcitonin [0–0.5 ng/mL]	1.9 (0.78–5.96)	3.96 (0.84–5.96)	1.73 (0.63–4.54)
Ferritin [13–68 ng/mL]	168 (109.5–271)	173 (107.6–337.5)	151.5 (113.5–220.75)
Leukocytes [4–12 × 10^3^/ µL]	17.32 (12.85–24.44)	16.66 (12.32–20.69)	15.29 (13.75–23.47)
Absolute lymphocytes [×10^3^/ µL]	1.58 (1.01–2.6)	1.18 (0.51–2.30)	2.54 (1.58–2.69)
Platelet count [×10^3^ /µL]	272.3 (209.5–592.4)	301.5 (188.7–563.7)	258.2 (241–609.5)
D-dimers [mg/L]	1.85 (0.8–5.28)	2.39 (0.9–9.46)	1.72 (0.71–2.68)
Fibrinogen [mg/mL]	443 (264.75–637)	478 (294.25–656.5)	463 (255.5–642)
Hemoglobin [g/dL]	10.78 (9.98–11.9)	10.78 (9.8–11.8)	10.8 (10.4–11.1)
Ionic calcium [mg/dL]	4.23 (4–4.36)	4.18 (3.9–4.34)	4.3 (4.16–4.37)
Creatinine [mg/dL]	0.31 (0.24–0.46)	0.32 (0.23- 0.43)	0.29 (0.26–0.49)
Seric sodium [mmol/L]	135 (132.5–136.5)	134.5 (133–135)	135 (132–138)
Seric potassium [mmol/L]	3.96 (3.64–4.8)	3.92 (3.19–4.33)	4.4 (3.91–4.96)
Bicarbonate (ECO_2_) [mmol/L]	22 (18–26.5)	25 (18–31)	21 (19–24)
Total protein [g/dL]	6.3 (6–6.9)	6.3 (5.6–6.8)	6.4 (6.3–6.9)
Aspartate aminotransferase [U/L]	36 (26.2–52)	29 (22–51.5)	31 (24–39)
Alanine aminotransferase [U/L]	22 (16.5–40)	38 (30–61)	17 (15–20)
Urine density	1012 (1007–1023)	1011.5 (1007–1024.75)	1013 (1008–1020)

**Table 4:** Demographic, clinical, and paraclinical characteristics hyponatremia, hypokalemia, hyperkalemia, metabolic acidosis, and metabolic alkalosis.

Variables	HipoNa N = 17	HipoK N = 10	HiperK N = 8	Metabolic Acidosis N = 4	Metabolic Alkalosis N = 10
**Categorial variables N (%)**					
Female:male ratio	1:1.12	1.5:1	3:1	1:3	1.5:1
Pediatric surgery (N = 31)	4 (12.9)	3 (9.7)	1 (3.2)	1 (3.2)	3 (9.7)
Admission into PICU (n = 17)	10 (58.8)	7 (41.1)	2 (11.7)	3 (17.6)	8 (47)
Severe-form MIS-C with H-E and A-B disorders	10 (58.8)	8 (80)	3 (37.5)	2 (50)	9 (90)
Moderate-form MIS-C with H-E and A-B disorders	7 (41.2)	2 (20)	5 (62.5)	2 (50)	1 (10)
**Numerical variables—Median (IQR)**					
Age (years)	9 (4–10)	7.5 (1.3–9)	1 (0.0–2.5)	7.5 (6.3–9.8)	8.5 (4–13)
Duration of hospitalization (days)	8.5 (5.5–11.5)	12.5 (11.5–15)	8 (6.8–11.3)	9 (6.8–11.8)	12 (9.3–15)
**Symptoms N (%)**					
Fever	17 (100)	10 (100)	7 (87.5)	3 (75)	3 (30)
Cough, dyspnea	3 (17.6)	2 (20)	2 (25)	3 (75)	4 (40)
Loss of appetite	8 (47)	6 (60)	6 (75)	3 (75)	3 (30)
Abdominal pain	6 (35.3)	3 (30)	3 (37.5)	3 (75)	4 (40)
Vomiting	6 (35.3)	7 (70)	7 (87.5)	3 (75)	4 (40)
Diarrhea	5 (29.4)	1 (10)	1 (12.5)	2 (50)	2 (20)
Skin rash	10 (58.9)	3 (30)	3 (37.5)	0 (0.0)	4 (40)
Oral thrush	4 (23.5)	3 (30)	3 (37.5)	0 (0.0)	1 (10)
Conjunctival hyperemia	3 (17.6)	2 (20)	2 (25)	0 (0.0)	1 (10)
Microadenopathy	4 (23.5)	3 (30)	3 (37.5)	3 (75)	0 (0.0)
Edema	5 (29.4)	2 (20)	0 (0.0)	0 (0.0)	0 (0.0)
Polyuria	4 (23.5)	3 (30)	3 (37.5)	0 (0.0)	3 (30)
**Biological markers—Median (IQR)**					
C-reactive protein [mg/dL]	15.71 (7.62–25.67)	24.51 (9.23–28.71)	22.99 (4.36–26.72)	24.74 (17.34–31/64)	10.41 (4.49–25.1)
Erythrocyte sedimentation rate [mm/h]	75 (30–94)	66.5 (34–79.2)	64 (23.5–100)	80 (57–103)	30.5 (24.7–66)
Procalcitonin [ng/mL]	3.4 (1.1–6.1)	3.7 (1.3–6.5)	3.9 (0.7–7.8)	1.3 (0.8–2.6)	1.3 (0.5–6.5)
Absolute lymphocytes [×10^3^/µL]	1.1 (0.4–1.7)	1.3 (0.3–1.7)	2.8 (2.6–3.3)	1.3 (0.9–1.7)	1.3 (0.3–2.1)
Platelet count [×10^3^/µL]	256 (209.4–308.5)	227.1 (125.5–443.9)	531.9 (345.2–720.6)	277.8 (181.9–528.9)	454.4 (134.5–636.8)
Seric sodium [mmol/L]	133 (132–134)	133.5 (132.3–135)	137 (135–139.5)	133.5 (132.8–135)	135 (133.3–137.5)
Seric potassium [mmol/L]	3.91 (3.2–4.1)	3.16 (2.9–3.58)	5.2 (5–5.34)	3.9 (3.8–3.9)	4.3 (2.9–4.6)
Bicarbonate (ECO_2_) [mmol/L]	22 (18–31)	19.5 (17–31)	22.5 (20.8–25.8)	15 (13.5–16)	31.5 (28.8–33)

## Data Availability

Personal medical data are publicly unavailable due to privacy and ethical restrictions, having been obtained from the medical records of patients admitted to the Emergency Clinical Hospital for Children “Sf. Ioan”, Galati.

## Abbreviations

A-B	Acid–base
ADH	Antidiuretic hormone
CDC	Centers for Disease Control and Prevention
COVID-19	Coronavirus Disease 2019
CRP	C-reactive protein
CTSE	Council of State and Territorial Epidemiologists
ESR	Erythrocyte sedimentation rate
H-E	Hydroelectrolyte
MIS-C	Multisystem inflammatory syndrome in children, associated with COVID-19
PICU	Pediatric intensive care unit
R.A.	Alkaline reserve (synonym ECO_2_-bicarbonate)
RT-PCR	Real-time polymerase chain reaction
SARS-CoV-2	Severe Acute Respiratory Syndrome-Related Coronavirus 2
WHO	World Health Organization
